# Quantum Crystallography: Exploring Electron Density and Interactions

**DOI:** 10.1063/4.0000821

**Published:** 2025-10-27

**Authors:** Sylwia Pawledzio, Xioping Wang

**Affiliations:** Neutron Scattering Division, Oak Ridge National Laboratory, Oak Ridge, TN, USA

## Abstract

Quantum Crystallography (QC) is an evolving field that integrates quantum mechanics with crystallographic data analysis to achieve a more accurate and detailed description of molecular and solid-state structures. Unlike conventional refinement methods, which rely on spherical atomic scattering factors and empirical constraints, QC incorporates theoretical electron densities or wavefunctions to enhance structural modeling. This approach not only improves the accuracy of atomic positions and thermal parameters but also provides deeper insights into electronic structures, intermolecular interactions, and charge density distributions ^1,2^

In this talk, I will discuss the fundamental differences between the traditional spherical a .model and aspherical refinement, emphasizing the growing importance of the latter in crystallography. QC encompasses a range of advanced refinement techniques that extend beyond conventional methods, offering enhanced structural insights. I will provide an overview of the QC framework, covering aspherical approaches based on experimental electron density, wavefunction-based refinements, databank-driven refinements, and extremely localized molecular orbitals, along with available computational tools for their implementation^3–9^.

A key focus will be on the practical aspects of QC, including multipolar refinement^3^ and Hirshfeld Atom Refinement (HAR)5. I will explain the concept of aspherical scattering factors, walk through example input files, and demonstrate how to set up and run relevant software, with particular emphasis on the importance of high-quality experimental data in achieving meaningful results.

Modern applications of QC will also be explored, including relativistic HAR^10^, spin density refinement^11^, the study of metallophilic interactions^12^, and the use of Transferable Aspherical Atom Model (TAAM) refinements to reconstruct electron densities in pharmaceutical research, such as NSAID interactions^13^. Additionally, I will highlight the potential of QC for studying direct air capture (DAC) materials^14^, with an emphasis on the integration of neutron and X-ray diffraction techniques to provide a comprehensive understanding of hydrogen-bonding interactions and charge density distributions.

By presenting both theoretical concepts and practical applications, this talk will offer a clear perspective on how QC methods can enhance structural refinement. Attendees will gain a deeper understanding of the advantages and limitations of different approaches, equipping them with the knowledge to assess when and how to apply QC techniques in their own research.
